# Boiled Down Poets

**Published:** 1910-12

**Authors:** 


### Boiled Down Poets.

How they would appear after the "Copy Editor" fixed them.

Miss B. Frietsche, a spinster of Frederick, Md., narrowly escaped being killed this morning. A troop of Confederates, General T. J. Jackson commanding, were marching down Fourth Street, when Miss Frietsche unfurled a flag from her attic window. The men were about to shoot when General Jackson ordered them to desist, under pain of ignominous death.

George H. Cassablanca, fifteen years of age, was burned on a steamer this morning. The vessel was burning and young Cassablanca refused to leave it before being ordered so to do by his father, who, however, had already perished. The boat was demolished, partly covered by insurance.

Three fishermen, all married, who sailed away at sundown last evening were drowned during the night. The bodies were found early this morning.

Edgar Wilson Nye, a newspaper man, assaulted Ah Sin, a Chinaman, here late last night, in an altercation over a euchre game. Nye was under the impression, he alleged, that the Chinaman had attempted to cheat. He declares that twenty-four packs of playing cards were found in the Mongolian's long sleeves and that his nails were waxed. Nye was discharged.

(Notes furnished by the Printer's Devil.)

1. Whittier's Barbara Fritsche.
2. The Boy Stood on the Burning Deck.
3. Chas. Kingsley's Three Fishers went sailing out on the sea.
4. Bret Harte's "Plain Language From Truthful James."—*Indianapolis Medical Journal.*

---

Loud buzzing in one ear should make one suspect arteriovenous aneurism at once.—*American Journal of Surgery.*

